# Augmented reality-based fine arts education experiences and adolescents’ perceived reduction in depressive symptoms: a self-report study of associations with psychological resilience, rumination, and emotional regulation

**DOI:** 10.3389/fpsyt.2026.1888424

**Published:** 2026-07-06

**Authors:** Da Wang, Rui Ye, Kaiwen Ren, Chengrui Gao

**Affiliations:** 1 Graduate School of Fine Arts, Hongik University, Seoul, Republic of Korea; 2 School of Fine Arts, Northwest Minzu University, Lanzhou, China; 3 School of Smart Education, Jiangsu Normal University, Xuzhou, China; 4 College of Computer Science, Sichuan University, Chengdu, China

**Keywords:** AR-based fine arts education, emotional regulation, perceived reduction in depressive symptoms, perceived reduction in rumination, psychological resilience

## Abstract

As depressive symptoms among adolescents continue to rise, increasing attention has been directed toward educational contexts that may support psychological well-being. Augmented reality, characterized by immersive, interactive, and multisensory affordances, has been increasingly incorporated into fine arts education. However, it remains insufficiently understood how adolescents’ perceived AR-based fine arts education experiences are associated with psychological resilience, emotional regulation, perceived reduction in rumination, and perceived reduction in depressive symptoms. Using post-course self-report questionnaire data from 518 Chinese adolescents, this study tested a cross-sectional associational model with structural equation modeling, without making causal or clinical claims. The empirical results indicate that adolescents’ perceived AR-based fine arts education experiences were positively associated with self-reported perceived reduction in depressive symptoms. Additionally, these experiences were positively associated with self-reported psychological resilience and perceived reduction in rumination. Self-reported psychological resilience and perceived reduction in rumination were positively associated with self-reported emotional regulation, and this regulation was positively associated with self-reported perceived reduction in depressive symptoms. These findings support a correlational psychological model linking perceived AR-based fine arts education experiences with adolescents’ self-reported psychological perceptions and perceived reduction in depressive symptoms. Theoretically, this study contributes to the literature on arts education, educational technology, and adolescent mental health. Practically, the findings suggest that immersive art-learning environments may be perceived by adolescents as supportive contexts for psychological resources. The innovation of this study lies in reframing AR-based fine arts education experiences as psychologically meaningful educational experiences associated with adolescents’ self-reported psychological perceptions

## 1 Introduction

Adolescent depressive symptoms are a significant mental health concern worldwide (1), particularly in China, where rapid social, cultural, and technological changes are associated with young people’s psychological well-being (2, 3). The prevalence of depressive symptoms among adolescents has received increasing attention in recent decades, and recent studies suggest an upward trend in self-reported depressive symptoms among Chinese adolescents (4, 5). These symptoms are often characterized by persistent feelings of sadness, loss of interest, and a range of emotional and cognitive difficulties that can interfere with daily functioning (6). Unaddressed depressive symptoms during adolescence may be associated with academic difficulties, strained social relationships, and an increased risk of later psychological problems, including suicidal ideation and behavior (7). Given these concerns, it is important to explore school-based educational experiences that may be associated with adolescents’ psychological well-being and their perceived reduction in depressive symptoms in China (8–10).

In recent years, various psychological and educational approaches have been examined in relation to adolescent emotional well-being, with art education emerging as a promising educational context. Previous studies have highlighted the psychosocial relevance of art activities for supporting emotional expression and supporting adolescents’ emotional well-being in adolescents (11, 12). Specifically, engaging in creative processes such as drawing, painting, and sculpting has been shown to help adolescents externalize their inner emotions, promote self-reflection, and support mood regulation (13). Furthermore, art-based educational activities have been associated with social skills, self-esteem, and overall mental well-being, all of which are critical factors related to adolescents’ psychological adjustment (14, 15).

While traditional art education has been linked to positive emotional and developmental outcomes, the integration of innovative technologies such as augmented reality (AR) offers additional layers of engagement and interaction that may enrich students’ learning and emotional experiences. AR technology blends digital elements with the real world, providing immersive and interactive learning experiences that have been shown to increase engagement and motivation in adolescents (16). In the context of fine arts education, AR allows students to interact with 3D art objects, visualize creative processes in real time, and receive instant feedback, all of which can enhance both the learning experience and students’ opportunities for emotional expression, reflection, and meaning-making (17, 18).

The application of AR in psychology-related and health-oriented educational contexts has gained increasing attention because of its potential to create immersive, interactive, and emotionally engaging experiences (19). AR has been utilized in a range of health-related and psychoeducational applications, including exposure-based applications for anxiety and phobias, as well as cognitive rehabilitation in patients with neurological disorders (20). In the context of adolescent depressive symptoms, AR’s ability to create engaging and interactive experiences may provide adolescents with a more dynamic educational medium for emotional expression, self-exploration, and reflective engagement (21, 22). Additionally, AR may support the development of resilience and coping-related experiences by providing safe and interactive environments for emotional exploration and learning (23).

Previous studies on arts-based education and creative expression have primarily focused on the emotional and psychological benefits of creative expression for adolescents, demonstrating that art education provides a safe space for emotional release, reducing stress and anxiety (such as: 24–26). However, despite these valuable insights, much of the existing research has not explored the roles of key psychological variables such as psychological resilience, perceived reduction in rumination, and emotional regulation in the context of art education and adolescent depressive symptoms. These variables are crucial to understanding how educational experiences may be associated not only with perceived symptom reduction but also with psychological processes relevant to adolescent well-being. Psychological resilience is vital because it reflects an adolescent’s capacity to adapt to stress and adversity, fostering recovery and maintaining emotional stability (27). Perceived reduction in rumination, on the other hand, is important for understanding adolescents’ subjective experience of disengaging from negative, repetitive thought patterns that are strongly linked to the development and persistence of depressive states (28). Finally, emotional regulation enables individuals to effectively manage and balance emotional responses, which is fundamental for understanding emotional dysregulation that often accompanies or exacerbates depressive symptoms (29).

In addition, much of the existing research has not incorporated modern technologies such as AR. While some studies have explored the use of digital tools in education, such as virtual reality for emotional regulation (21, 30), limited evidence is available on AR-based fine arts education in relation to adolescents’ self-reported depressive symptoms and related psychological constructs. More specifically, prior research has rarely examined how adolescents’ perceived AR-based fine arts education experiences are structurally associated with self-reported psychological resilience, self-reported perceived reduction in rumination, self-reported emotional regulation, and self-reported perceived reduction in depressive symptoms within a single theoretical model.

It is important to clarify the positioning of the present study. This study is not an intervention study and does not include a control group, random assignment, or a pretest-posttest design. Instead, it uses self-reported survey data from adolescents and applies structural equation modeling to examine the associations among perceived AR-based fine arts education experiences, self-reported psychological resilience, self-reported perceived reduction in rumination, self-reported emotional regulation, and self-reported perceived reduction in depressive symptoms. Accordingly, the purpose of this study is not to evaluate the clinical or therapeutic effectiveness of AR-based fine arts education, but to understand, from an educational psychology perspective, how immersive art-learning experiences may be related to adolescents’ subjective psychological improvement. In this sense, the study provides a theoretically grounded and empirically informed basis for future longitudinal, quasi-experimental, and intervention-oriented research.

The innovation of this study lies in its focus on these underexplored psychological constructs within the framework of AR-based fine arts education. While art-based activities have been associated with emotional and reflective benefits, the inclusion of AR technology may provide more immersive and engaging educational experiences that are meaningfully associated with psychological resilience, perceived reduction in rumination, and emotional regulation. By examining these psychological constructs, this research offers a more comprehensive understanding of how adolescents’ perceived AR-based fine arts education experiences are associated with adolescents’ self-reported psychological resilience, self-reported rumination-related perceptions, self-reported emotional regulation, and self-reported perceived reduction in depressive symptoms. This research addresses a gap in the literature by emphasizing the roles of resilience, perceived reduction in rumination, and emotional regulation in understanding adolescents’ perceived reduction in depressive symptoms within AR-supported fine arts education contexts.

Theoretically, this study contributes to the development of an integrative model that combines fine arts education, AR technology, and adolescent psychological development. It provides insights into how emerging technologies may be incorporated into school-based educational practices that are relevant to adolescent psychological well-being. Practically, this research may inform educators, school counselors, curriculum designers, and policymakers about the potential value of AR-supported fine arts education for creating engaging, reflective, and psychologically supportive learning environments for adolescents. The findings may provide preliminary evidence for considering AR-supported fine arts education as a promising educational context that may be relevant to adolescent psychological well-being and arts education for adolescents in China and beyond.

## 2 Literature review and research hypotheses

Based on existing theories and prior empirical studies, this section develops the research hypotheses regarding the theoretically expected associations among the constructs. All focal variables in this study were measured using adolescents’ self-reported survey responses. For readability, the construct names are used directly in the following hypotheses, but the proposed relationships should be understood as associations among self-reported measures.

### 2.1 AR-based fine arts education experiences and perceived reduction in depressive symptoms

Research into the intersection of mental health, arts-based education, and emerging technologies has revealed promising avenues for understanding adolescents’ emotional and psychological well-being. Arts-based activities and creative expression have been widely discussed for their relevance to emotional expression, self-reflection, and psychological adjustment (31, 32), and prior research on creative expression provides useful theoretical insights for understanding how art-learning experiences may relate to adolescents’ perceived psychological well-being.

The integration of AR into fine arts education presents an innovative approach to enriching students’ artistic, emotional, and reflective learning experiences. AR’s immersive, interactive, and multisensory capabilities offer a novel platform for engaging adolescents in creative expression (21, 33). By providing a dynamic, real-time environment in which adolescents can explore and process emotions through art, AR may support emotional engagement, attentional involvement, and reflective meaning-making (34). Furthermore, social cognitive theory (35) emphasizes the importance of self-efficacy and environmental interaction in shaping psychological functioning. AR-based fine arts education, by offering personalized experiences and fostering greater agency in the creative process, may be associated with adolescents’ perceived sense of control and self-efficacy, which are relevant to psychological adjustment and well-being (36).

Additionally, AR’s ability to provide immediate feedback and adapt to an individual’s progress may foster a sense of achievement and competence. This immediate reinforcement may be relevant to adolescents’ self-esteem, perceived competence, and positive emotional engagement. As research indicates, positive reinforcement can strengthen emotional resilience, enabling adolescents to confront negative emotions more effectively (34, 37). Within an educational context, such experiences may help adolescents perceive greater emotional relief and psychological improvement.

Given the increasing interest in digital learning environments and technology-supported well-being contexts, particularly those involving adolescents, AR-based fine arts education may provide a promising educational context for examining adolescents’ perceived psychological improvement. The potential for AR to create an immersive, engaging environment that fosters emotional processing and reflective engagement provides a theoretical basis for expecting a positive association between AR-based fine arts education experiences and adolescents’ perceived reduction in depressive symptoms (15, 22).

Based on the theoretical foundations and empirical evidence discussed above, the following research hypothesis is proposed:

H1: AR-based fine arts education experiences are positively associated with adolescents’ perceived reduction in depressive symptoms.

### 2.2 AR-based fine arts education experiences and psychological resilience

The integration of AR into fine arts education offers a novel avenue for examining psychological resilience among adolescents (38). Psychological resilience refers to an individual’s ability to adapt to stressors, adversity, or trauma and is a critical factor in mental health, particularly for adolescents, who are vulnerable to emotional and psychological challenges during developmental stages (39).

Fine arts education, particularly when facilitated through innovative technologies like AR, may be associated with cognitive and emotional processes that foster resilience (40, 41). According to theories of emotional regulation (42), engaging in creative activities facilitates emotional expression and regulation, which is a central component of resilience. Through AR-based fine arts education, adolescents are provided with immersive, interactive experiences that stimulate both cognitive and emotional engagement, thereby offering opportunities for emotional expression, perspective-taking, and adaptive reflection (38, 40). The dynamic interaction between the digital and physical world may also strengthen adolescents’ perceived agency and mastery in the learning process, both of which are theoretically relevant to resilience.

Based on the theoretical foundations and empirical evidence discussed above, the following research hypothesis is proposed:

H2: AR-based fine arts education experiences are positively associated with adolescents’ psychological resilience.

### 2.3 AR-based fine arts education experiences and perceived reduction in rumination

Rumination refers to the repetitive and passive focus on distressing thoughts, which has been consistently linked to various mental health conditions, particularly depression and anxiety (43). According to the self-regulation model of rumination, individuals who engage in excessive rumination are prone to intensifying negative affective states and experiencing difficulties in emotional regulation (44). This cycle can aggravate depressive symptoms and hinder effective coping strategies. In this context, addressing rumination is essential for understanding psychological vulnerability and emotional well-being, particularly in adolescents, who are at heightened risk for both rumination and depressive symptoms (45).

Art-based educational activities, particularly those incorporating modern technologies such as AR, offer novel opportunities to engage individuals in creative and cognitive processes that can shift attention, support emotional expression, and facilitate reflective processing (46). The hypothesis posits that AR-based fine arts education provides a dynamic platform for adolescents to interact with their emotions, potentially promoting their perceived reduction in rumination by encouraging cognitive flexibility, emotional expression, and mindful engagement with the present moment.

The underlying mechanisms by which AR-based fine arts education may be associated with perceived reduction in rumination are rooted in cognitive and emotional processes. AR technology, by virtue of its interactive and immersive nature, engages multiple senses and captures attention, redirecting adolescents’ focus away from repetitive, negative thoughts (21). Previous studies have shown that mindfulness-based approaches, which emphasize present-moment awareness, are effective in reducing rumination (47). AR fine arts education may share some features with present-focused engagement by allowing adolescents to experience a more engaging, immersive learning environment that fosters concentration and reduces cognitive distractions (21). Additionally, the creative expression inherent in art education—when combined with AR’s immersive capabilities—may serve as a form of emotional processing (48). This process allows adolescents to externalize and symbolically represent internal emotional states, potentially helping them perceive a reduction in repetitive negative thinking through symbolic expression and reflective reappraisal.

In theoretical terms, cognitive behavioral therapy (CBT) (49) has long been used to target rumination by helping individuals identify and challenge negative thought patterns. Although AR-based fine arts education should not be equated with CBT or clinical treatment, it may provide educational experiences that encourage adolescents to shift attention from repetitive negative thoughts toward creative, constructive, and present-focused activities. Additionally, self-determination theory (50) suggests that psychological needs for autonomy, competence, and relatedness must be met for individuals to experience well-being and emotional regulation. By supporting these needs in a creative and interactive setting, AR-based art education may foster a sense of agency and self-efficacy that is theoretically related to adolescents’ perceived reduction in rumination.

Therefore, the following research hypothesis can be inferred:

H3: AR-based fine arts education experiences are positively associated with adolescents’ perceived reduction in rumination.

### 2.4 Psychological resilience and emotional regulation

Psychological resilience plays a pivotal role in understanding emotional regulation, particularly in adolescents who are vulnerable to psychological stressors such as depressive symptoms (51). Psychological resilience is defined as the capacity to adapt to adversity, and research has consistently shown its strong association with emotional regulation, a key component of mental health (52). Adolescents with higher psychological resilience tend to employ more adaptive coping strategies, which help regulate their emotional responses to distressing situations (53). This adaptive regulation is especially crucial in the context of depressive symptoms, where poor emotional regulation can lead to emotional dysregulation, exacerbating symptoms of depression and contributing to a cycle of negative affect (54). Thus, resilience is theoretically relevant to adolescents’ capacity to manage emotional responses and maintain psychological adjustment.

The mechanism by which psychological resilience is associated with emotional regulation can be explained through several psychological models. According to the transactional model of stress and coping (55), individuals’ emotional responses to stress are shaped by their appraisal of the stressor and their perceived ability to cope with it. In this framework, resilience is conceptualized as a resource that enables individuals to evaluate stressors more positively and engage in more effective coping strategies (56). These strategies include emotional reappraisal, which has been linked to improved emotional regulation (57). In adolescents, resilient individuals are more likely to regulate negative emotions such as sadness or anxiety, thus supporting more adaptive emotional functioning (58). Furthermore, research on self-efficacy (59) suggests that resilient individuals demonstrate higher confidence in their ability to manage challenging emotions, further supporting the expected association between resilience and emotional regulation.

Therefore, the following research hypotheses can be inferred:

H4: Psychological resilience is positively associated with adolescents’ emotional regulation.

### 2.5 Perceived reduction in rumination and emotional regulation

Rumination, defined as the repetitive and passive focus on distressing thoughts, is a well-established risk factor for emotional dysregulation, particularly in adolescents experiencing depressive symptoms (60). Research has demonstrated that rumination exacerbates emotional distress and impairs the ability to regulate emotions effectively, thereby increasing the likelihood of prolonged depressive episodes (45). In contrast, lower levels of rumination, or adolescents’ perceived reduction in rumination, may be related to more adaptive emotional regulation and psychological well-being (61). Adolescents who exhibit reduced levels of rumination are more likely to adopt adaptive emotional regulation strategies, such as cognitive reappraisal and mindfulness, which can mitigate the intensity and duration of negative emotional states (62). Thus, perceived reduction in rumination is expected to be positively associated with emotional regulation.

The mechanism by which rumination influences emotional regulation can be explained through the cognitive theory of emotion (63), which suggests that maladaptive cognitive processes, such as rumination, interfere with an individual’s ability to modulate emotional responses. Rumination heightens negative cognitive biases and hinders the reappraisal of emotional stimuli, leading to sustained emotional distress (62). When rumination is reduced, adolescents are better able to engage in more adaptive emotion-regulation strategies, such as reappraisal, which involves reinterpreting the meaning of distressing situations in a more positive light (64, 65). Furthermore, research suggests that decreasing rumination allows for more flexible emotional responses, enabling adolescents to manage negative emotions more effectively (66). By interrupting the cycle of negative thinking and emotional dysregulation, rumination reduction fosters a more resilient emotional response, which is essential for maintaining mental health. In the present study, this relationship is examined at the level of adolescents’ perceived reduction in rumination rather than objectively measured change in rumination.

Therefore, the following research hypotheses can be inferred:

H5: Perceived reduction in rumination is positively associated with emotional regulation in adolescents.

### 2.6 Emotional regulation and perceived reduction in depressive symptoms

From a theoretical standpoint, emotional regulation is a core process in the understanding of depressive symptoms and adolescent psychological well-being, particularly in adolescents, whose emotional experiences are often intense and difficult to control (67). Emotional regulation theory (68) posits that individuals employ a range of strategies to influence the intensity, duration, and expression of their emotional responses. In adolescents with depressive symptoms, these strategies are frequently maladaptive, which may amplify negative emotional states (69). The cognitive emotional regulation framework (70) suggests that when adolescents are able to use adaptive strategies like cognitive reappraisal, which involves changing the way one interprets emotionally charged situations, they can reduce the intensity of negative emotions and, thereby, experience lower levels of depressive affect or perceive improvement in depressive symptoms (68). These studies highlight that effective emotional regulation serves as a protective factor against the onset and persistence of depression, supporting the theoretical relevance of emotional regulation to adolescents’ perceived psychological improvement (71, 72).

Further, the self-regulation model (73) posits that the ability to manage one’s emotional and cognitive responses is influenced by an individual’s capacity for self-control and self-awareness. Adolescents with depressive symptoms often struggle with both self-control and emotional awareness, leading to a vicious cycle where unregulated emotions contribute to the persistence of depressive states (74). Improving emotional regulation, therefore, is theoretically associated with greater emotional flexibility, better cognitive reappraisal, and adolescents’ subjective perceptions of psychological improvement. The theory of cognitive vulnerability (75) also suggests that individuals with depression tend to exhibit negative biases in their emotional processing, interpreting ambiguous stimuli as threatening or overwhelming. By using more adaptive emotional regulation, adolescents can reframe these emotional experiences more adaptively, thus potentially perceiving fewer depressive symptoms or greater relief from negative emotional states (76).

Therefore, can put forward the following hypothesis:

H6: Emotional regulation is positively associated with adolescents’ perceived reduction in depressive symptoms.

**Figure 1:**
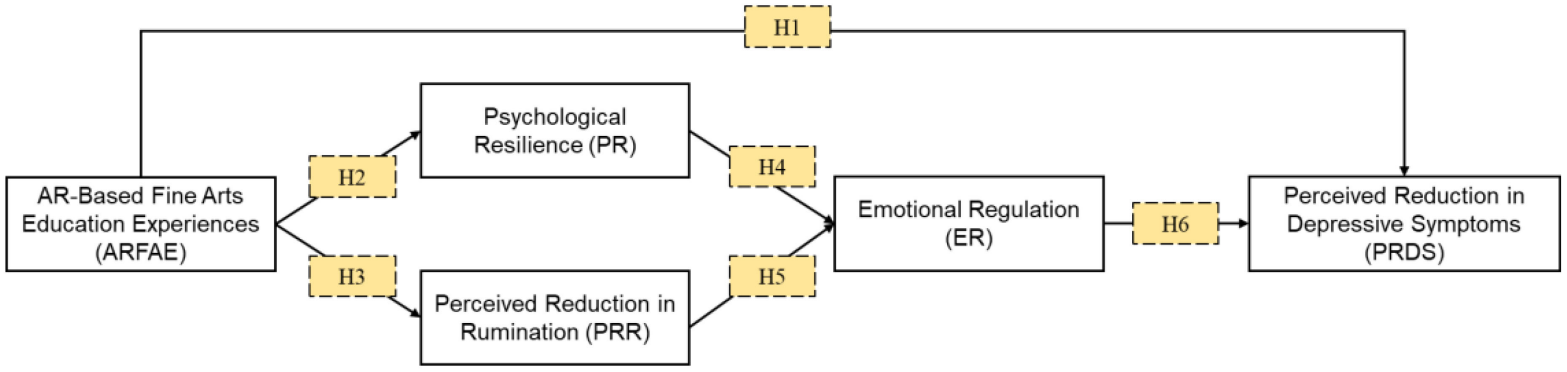
Research hypotheses and model.

In conclusion, **Figure 1** presents the research hypotheses and model of this study.

## 3 Methodology

### 3.1 Research design and positioning

This study adopted an observational, cross-sectional survey design to examine adolescents’ perceived experiences after participating in an AR-based fine arts education course and their associations with self-reported psychological constructs. The study was positioned as educational research rather than a clinical intervention trial. Accordingly, the AR-based fine arts course was conceptualized as a school-based educational experience that integrated augmented reality technology, visual art creation, emotional expression, individual reflection, and peer collaboration. It was not designed or evaluated as psychotherapy or as a controlled mental health intervention.

The central explanatory construct in this study was AR-Based Fine Arts Education Experiences (ARFAE), which referred to students’ perceived immersion, interactivity, creative engagement, emotional expression, and reflective participation during the course. The study examined the relationships among ARFAE, Psychological Resilience (PR), Perceived Reduction in Rumination (PRR), Emotional Regulation (ER), and Perceived Reduction in Depressive Symptoms (PRDS). The questionnaire therefore measured adolescents’ self-reported course experiences and psychological perceptions after course participation.

This design was considered appropriate because the study focused on adolescents’ subjective educational experiences in a real-world school-based AR fine arts learning context. A survey-based design allowed data to be collected from a relatively large adolescent sample and enabled the examination of theoretically informed associations among multiple constructs. Structural equation modeling was used to test the proposed relationships among ARFAE, PR, PRR, ER, and PRDS. However, because the data were collected at a single time point and the study did not include a pretest, control group, or random assignment, the findings were interpreted as associations among self-reported constructs rather than as evidence of causal effects or long-term intervention outcomes.

Overall, by positioning the course as an educational experience and the data as self-reported post-course perceptions, this study maintained consistency among its research design, measurement strategy, analytical approach, and interpretation of findings. This positioning allowed the study to examine the psychological relevance of AR-based fine arts education for adolescents while avoiding unsupported causal or clinical claims.

### 3.2 Course design

The AR-based fine arts education course was implemented as a school-based art-learning program that integrated augmented reality technology with creative visual expression. The course was not designed as a clinical intervention or psychotherapy program. Rather, it provided an immersive educational context in which adolescents could engage in art creation, emotional expression, collaborative learning, and reflective activities. In line with the objectives of the present study, the course context was theoretically relevant to psychological resilience, perceived reduction in rumination, emotional regulation, and perceived reduction in depressive symptoms. **Table 1** presents the overall framework of the course design.

**Table 1:** Course design and content.

Week	Session title	Description
Week 1	Introduction to AR and Self-Expression	Students were introduced to the basic functions of AR applications used for creating and manipulating three-dimensional visual artworks. They then created an initial AR artwork representing their current emotional state. This session familiarized students with the technological tools and established a foundation for subsequent creative expression.
Week 2	Transforming Emotions	Students explored how emotions could be visually represented and transformed through AR-based artistic techniques. They created artworks depicting a negative emotional experience and then modified the visual features of the artwork through AR effects. This activity encouraged students to represent emotional experiences symbolically and to consider alternative interpretations of negative feelings.
Week 3	Navigating Challenges	Students participated in AR-supported storytelling activities. They created visual scenes representing personal or everyday challenges and placed these scenes within digital environments. Through this activity, students were encouraged to articulate challenges through artistic expression and to reflect on possible ways of responding to difficulties.
Week 4	Building Resilience	Students created artworks symbolizing personal strengths, coping resources, and supportive relationships. AR overlays were used to add virtual elements representing sources of encouragement, such as peers, family members, teachers, or mentors. This session provided opportunities for students to reflect on internal and external resources that may be relevant to resilience.
Week 5	Reflecting on Repetitive Negative Thoughts	Students used AR tools to externalize repetitive negative thoughts through a visual mapping activity. For example, they created a “thought tree” in which different branches represented recurring negative thought patterns. By visualizing these thought patterns, students were encouraged to observe and describe their thinking processes from a more distanced perspective. This session was conceptually related to perceived reduction in rumination.
Week 6	Emotional Regulation Through Creative Environments	Students created calming AR landscapes that represented feelings of safety, balance, or calmness. During the session, they were guided to reflect on how visual elements, colors, spatial arrangements, and symbolic images could be used to represent and regulate emotional experiences. The activity was designed to connect creative expression with adolescents’ understanding of emotional regulation in an educational setting.
Week 7	Connecting Through Art	Students worked in pairs or small groups to create collaborative AR artworks representing shared experiences or common themes. This session emphasized peer interaction, communication, and collective artistic creation. Through collaborative work, students had opportunities to experience social connection and mutual support within the art-learning environment.
Week 8	Collective AR Exhibition	Students presented their AR artworks in a class-based exhibition. The exhibition allowed students to share their creative processes, reflect on their learning experiences, and discuss how they represented emotions, challenges, strengths, and social connections through AR-based art. This final session served as a reflective conclusion to the course.

The course was delivered as an educational program rather than a therapeutic service. It was implemented by trained art educators and research facilitators who followed a standardized course protocol. No psychological diagnosis, counseling, psychotherapy, or individualized therapeutic treatment was provided during the course.

The course lasted eight weeks, with one 90-minute session each week. Each session was organized around an artistic theme and incorporated AR-supported visual creation, individual reflection, and peer interaction. The major themes included “Transforming Emotions,” “Navigating Challenges,” “Building Resilience,” and “Connecting Through Art.” These themes were selected to provide adolescents with opportunities to express emotions, represent personal experiences, explore coping-related ideas, and engage in collaborative artistic practices. The course emphasized educational engagement and creative exploration rather than clinical treatment.

To ensure procedural consistency, the same AR applications, instructional materials, activity guidelines, and session structure were used across participating classes. Before course implementation, instructors received training on the use of AR tools and the course protocol. Although the course themes were theoretically aligned with the constructs examined in the study, the course was not presented to students as a mental health intervention, and students were not informed of the specific hypothesized relationships among the research variables. **Table 1** shows the weekly course design and content details.

Overall, the AR-based fine arts education course provided an immersive and interactive learning environment in which adolescents engaged with artistic creation, emotional expression, reflective thinking, and peer collaboration. The course design was theoretically aligned with the psychological constructs examined in this study, including psychological resilience, perceived reduction in rumination, emotional regulation, and perceived reduction in depressive symptoms. However, given the observational and cross-sectional nature of the present study, the course is treated as an educational context rather than as a controlled psychological intervention.

### 3.3 Measurement scales

The questionnaire was designed to assess adolescents’ perceptions of their AR-based fine arts education experiences and their self-reported psychological characteristics after participating in the course. Consistent with the research objectives, the measurement instrument included five constructs: AR-Based Fine Arts Education Experiences (ARFAE), Psychological Resilience (PR), Perceived Reduction in Rumination (PRR), Emotional Regulation (ER), and Perceived Reduction in Depressive Symptoms (PRDS). Given the observational and cross-sectional nature of the study, the questionnaire was used to examine associations among these constructs rather than to establish the causal effectiveness of the course.

The measurement items were developed by adapting established scales from psychology, education, and technology-enhanced learning research. Items related to psychological resilience, rumination, emotional regulation, and depressive symptoms were adapted from validated instruments and reworded to fit the context of AR-based fine arts education. To ensure contextual relevance, all items were phrased in relation to students’ experiences after participating in the AR-based fine arts course.

During the adaptation process, the original items were not used to diagnose clinical symptoms or objectively measure psychological change. Instead, they were reworded to reflect adolescents’ self-reported psychological perceptions after participating in the AR-based fine arts course. For example, items were contextualized by adding phrases such as “after participating in the AR-based fine arts course” or “since participating in the AR-based fine arts course.” This modification was intended to align the measurement items with the post-course, self-report, and cross-sectional design of the study. The wording was also simplified to ensure that the items were understandable for adolescent respondents.

Among them, the ARFAE items were designed to capture adolescents’ perceived AR-supported fine arts learning experience through three theoretically relevant dimensions: conceptual clarity, immersion in the art-making process, and enhanced visual observation. These dimensions correspond to core affordances of AR technologies in fine arts education, where understanding artistic concepts, engaging in immersive art-making, and observing visual or procedural details are central learning experiences.

All items were measured using a five-point Likert scale, ranging from 1 = strongly disagree to 5 = strongly agree. Higher scores indicated stronger perceived AR-based fine arts education experiences, higher self-reported psychological resilience, greater self-reported perceived reduction in rumination, stronger self-reported emotional regulation, and greater self-reported perceived reduction in depressive symptoms. The specific measurement items and their sources are presented in **Table 2**.

**Table 2:** Measurement scales.

Factors	Measurement items	Sources
AR-Based Fine Arts Education Experiences (ARFAE)	ARFAE1: The AR-based fine arts course helped me understand artistic concepts more clearly.	77, 78
	ARFAE2: The AR-based fine arts activities made me feel immersed in the art-making process.	
	ARFAE3: The AR tools helped me observe details of the art-making process more clearly.	
Psychological Resilience (PR)	PR 1: After participating in the AR-based fine arts course, I am still able to stay calm when facing stressful situations.	79, 80
	PR 2: After participating in the AR-based fine arts course, I believe in my ability to cope with challenges in life.	
	PR 3: After participating in the AR-based fine arts course, I try to find ways to solve problems rather than give up.	
Perceived Reduction in Rumination (PRR)	PRR1: Since participating in the AR-based fine arts course, I spend less time dwelling on past negative experiences.	81
	PRR2: Since participating in the AR-based fine arts course, I find it easier to disengage from repetitive thoughts about negative events.	
	PRR3: Since participating in the AR-based fine arts course, my tendency to ruminate has decreased.	
Emotional Regulation (ER)	ER 1: After participating in the AR-based fine arts course, I am able to adjust my emotional responses according to different situations.	82, 83
	ER 2: After participating in the AR-based fine arts course, I feel that I can remain calm and rational in emotions like anger and sadness.	
	ER 3: After participating in the AR-based fine arts course, I am better able to regulate my emotions.	
Perceived Reduction in Depressive Symptoms (PRDS)	PRDS 1: After participating in the AR-based fine arts course, I felt that my depressive feelings had decreased.	84, 85
	PRDS 2: After participating in the AR-based fine arts course, I felt less helpless or discouraged.	
	PRDS 3: After participating in the AR-based fine arts course, I felt more interested in daily life or learning activities.	

To improve content validity and contextual appropriateness, the adapted questionnaire was reviewed by experts in adolescent psychology, educational psychology, educational technology, and arts education. The expert review focused on whether the adapted items were theoretically consistent with the intended constructs, whether the wording was appropriate for adolescent respondents, and whether the items adequately reflected the AR-based fine arts education context. Based on the expert comments, minor wording adjustments were made to improve clarity and reduce potential ambiguity.

Before the formal survey, a pilot test was conducted with 50 adolescents who shared similar characteristics with the target sample. The pilot test was used to examine the clarity, readability, and contextual appropriateness of the questionnaire items. Participants were invited to provide feedback on whether the wording was understandable, whether any items were confusing, and whether the items were relevant to their course experience. Based on their feedback, minor wording revisions were made to improve item clarity, age appropriateness, and consistency of expression. No construct was removed after the pilot test.

The reliability and validity of the scales were further evaluated in the main study. Internal consistency was assessed using Cronbach’s alpha and composite reliability. Construct validity was examined through confirmatory factor analysis, including factor loadings, average variance extracted, and discriminant validity tests. These procedures were used to determine whether the measurement model adequately represented the constructs included in the proposed structural model.

Overall, the measurement design was intended to provide contextually appropriate and psychometrically sound indicators for examining associations among adolescents’ perceived AR-based fine arts education experiences, self-reported psychological resilience, self-reported perceived reduction in rumination, self-reported emotional regulation, and self-reported perceived reduction in depressive symptoms.

### 3.4 Data collection

The AR-based fine arts education course was a stand-alone program developed specifically for this study, and participants were recruited from four schools in China. Schools were identified through collaboration with administrators and art teachers and selected based on technological readiness and willingness to host the course.

To ensure meaningful exposure to the AR-based fine arts education experience, only students who completed all eight weekly sessions were eligible to complete the post-course questionnaire. Attendance records maintained by the participating showed that all 540 students invited to the survey had completed the full eight-session course. No student formally withdrew from the course during the eight-week implementation period. Therefore, the final survey pool consisted only of adolescents who had received full exposure to the AR-based fine arts education experience.

The survey was administered two weeks after the completion of the AR-based fine arts education course. This interval was selected to allow participants to reflect on their course experiences with some temporal distance from the final session and to reduce potential recency effects associated with immediate post-course responses. Given the cross-sectional design of the study, the survey was used to assess adolescents’ self-reported perceptions and psychological characteristics after course participation, rather than to evaluate the causal or enduring effects of the course.

Data were collected using a standardized administration procedure. Before completing the questionnaire, participants received a brief explanation of the study purpose, the voluntary nature of participation, and the instructions for completing the survey. They were informed that there were no right or wrong answers and that they should respond based on their own perceptions and experiences. Participants completed the questionnaire independently under the supervision of the research team, who were available only to clarify procedural questions and did not influence participants’ responses.

All procedures were conducted in accordance with ethical standards for research involving adolescent participants. Because the participants were minors, informed consent was obtained from their legal guardians, and assent was obtained from the students before data collection. Participation was voluntary, and participants were informed that they could withdraw from the study at any time without penalty.

To protect participants’ privacy, the survey was administered anonymously. No personally identifiable information, such as names, student identification numbers, phone numbers, or home addresses, was collected. All questionnaire responses were used only for academic research purposes and were stored securely. The data were analyzed and reported in aggregate form to ensure confidentiality.

**Figure 2:**
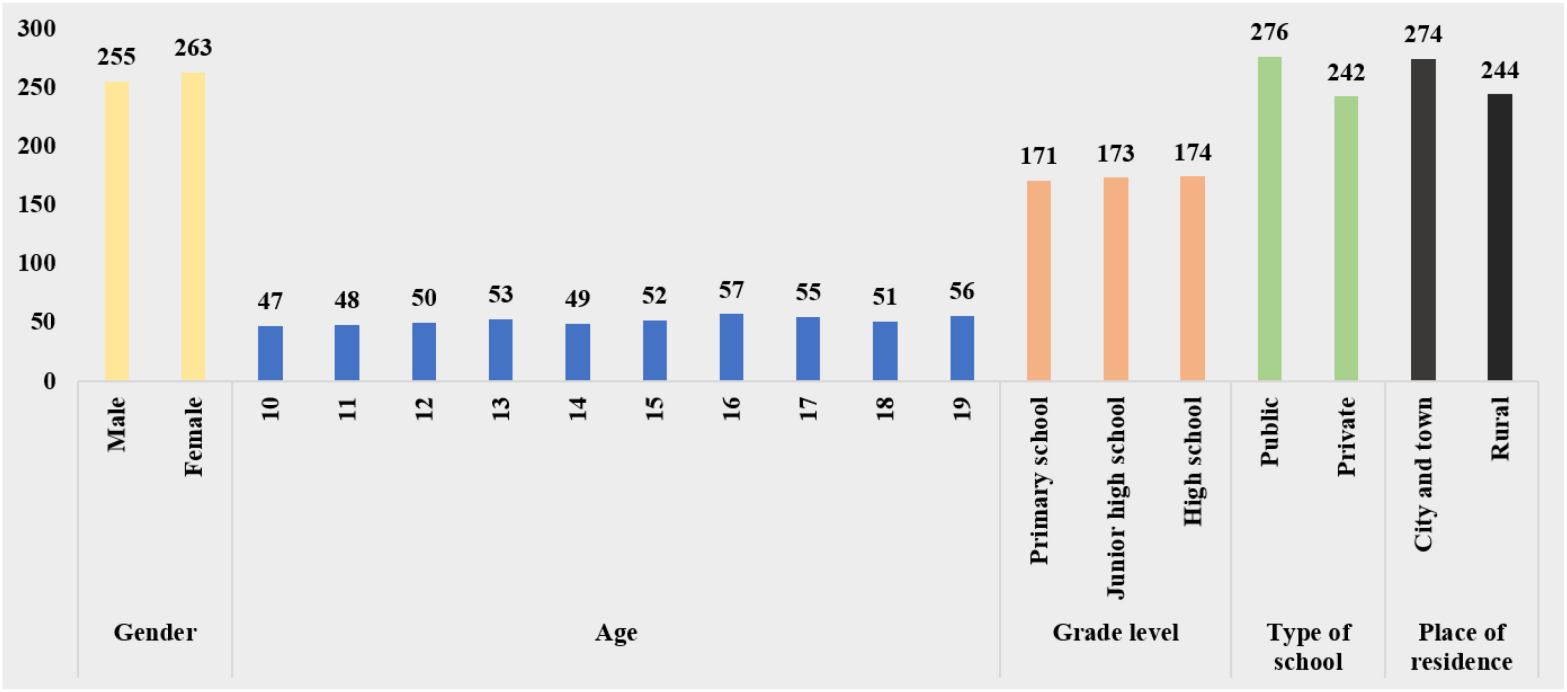
Information of 518 respondents.

A total of 540 participants were recruited and invited to complete the questionnaire. After data collection, returned questionnaires were screened according to predefined exclusion criteria. Questionnaires were excluded if they met any of the following conditions: missing responses, clear patterned responding such as selecting the same response option across nearly all items, or an unrealistically short completion time of less than three minutes. Based on these criteria, 22 questionnaires were excluded. Specifically, nine were excluded because of substantial missing data, seven because of patterned responding, and six because of unrealistically short completion times. After these exclusions, 518 valid questionnaires remained for SEM analysis. **Figure 2** presents the demographic information of the respondents.

### 3.5 Structural equation modeling (SEM)

Structural equation modeling (SEM) was chosen as the primary data analysis method in this study due to its ability to examine complex relationships among multiple latent variables, including AR-Based Fine Arts Education Experiences (ARFAE), Psychological Resilience (PR), Perceived Reduction in Rumination (PRR), Emotional Regulation (ER), and Perceived Reduction in Depressive Symptoms (PRDS), as well as their associated indicators. These constructs are multidimensional and cannot be directly observed, making SEM suitable for testing both the measurement model and the structural model within a single analytical framework. SEM allows for the evaluation of theoretically hypothesized structural paths while accounting for measurement error, thereby supporting a systematic examination of the associations among adolescents’ perceived AR-based fine arts education experiences and their self-reported psychological constructs. Given the cross-sectional design of this study, the SEM results were interpreted as associations rather than evidence of causal or intervention effects.

Demographic and contextual variables were not included as controls in the main SEM analysis because the study focused on testing the theoretically specified relationships among the core latent constructs, and no hypotheses were proposed regarding the effects of gender, age, grade level, school type, or place of residence. These variables were therefore reported only for sample description. Accordingly, the SEM results should be interpreted as unadjusted associations among ARFAE, PR, PRR, ER, and PRDS.

## 4 Empirical analysis

### 4.1 Assessment of measurement model

The empirical analysis was conducted using AMOS 26 and SPSS 31 software. Prior to the analysis, questionnaires containing missing values were removed; therefore, the final dataset used for SEM contained no missing data. The SEM models were estimated using the maximum likelihood estimation method.

To assess the potential influence of common method bias, this study first adopted Harman’s single-factor test, following the recommendation of Podsakoff et al. (86). The analysis indicated that the first factor extracted without rotation explained 27.786% of the total variance, which is considerably lower than the 50% criterion.

Moreover, an additional model comparison procedure was performed. A baseline model was first specified by constraining all observed indicators to load onto a single latent factor. Subsequently, a theoretically specified measurement model was estimated in which items were assigned to their corresponding constructs. The comparison between these two models was based on the differences in chi-square statistics and degrees of freedom. The results demonstrated that the theoretically specified model differed significantly from the single-factor model (Δχ² = 1618.389, Δdf = 10, p < 0.05), providing further evidence that common method bias was not a concern in this research (87).

The model fit indices show that chi-square/df is 2.929, GFI is 0.940, CFI is 0.956, SRMR is 0.043, TLI is 0.945, IFI is 0.956, and RMSEA is 0.061, indicating that the model fits well (88, 89).

**Table 3** shows the reliability findings for the measurement tools, and **Table 4** outlines the validity findings. According to Fornell and Larcker (90), an average variance extracted (AVE) of at least 0.5 is essential to confirm validity. Furthermore, this research utilized Cronbach’s alpha coefficient along with composite reliability measures to assess the validity of the questionnaire. A coefficient exceeding 0.7 suggests that the questionnaire possesses reliability. The tables illustrate that the internal consistency results for every instrument employed in this research are satisfactory (91).

**Table 3:** Reliability test.

Factors	Measurements	UnStd.	Std.	S.E.	Z
AR-Based Fine Arts Education Experiences (ARFAE)	AE1	1.000	0.733		
	AE2	1.042	0.749	0.077	13.449
	AE3	0.906	0.741	0.068	13.414
Psychological Resilience (PR)	PR1	1.000	0.810		
	PR2	0.923	0.804	0.054	17.155
	PR3	0.811	0.751	0.049	16.443
Perceived Reduction in Rumination (PRR)	IR1	1.000	0.741		
	IR2	1.124	0.743	0.079	14.260
	IR3	1.170	0.772	0.081	14.481
Emotional Regulation (ER)	ER1	1.000	0.703		
	ER2	1.180	0.824	0.078	15.045
	ER3	1.171	0.782	0.079	14.849
Perceived Reduction in Depressive Symptoms (PRDS)	IAD1	1.000	0.951		
	IAD2	1.080	0.891	0.032	33.670
	IAD3	0.996	0.875	0.031	32.134

**Table 4:** Validity test.

	Cronbach’s alpha	Average variance extracted (AVE)	Composite reliability (CR)
AR-Based Fine Arts Education Experiences (ARFAE)	0.783	0.549	0.785
Psychological Resilience (PR)	0.830	0.622	0.831
Perceived Reduction in Rumination (PRR)	0.794	0.566	0.796
Emotional Regulation (ER)	0.811	0.595	0.814
Perceived Reduction in Depressive Symptoms (PRDS)	0.929	0.821	0.932

Fornell and Larcker (90) highlighted that discriminant validity is established when the average variance extracted (AVE) for each construct is greater than the variance shared with other constructs. This can be illustrated by the square root of the AVE being higher than the correlation coefficients, as depicted in **Table 5**, demonstrating that the measurement items possess strong discriminant validity.

**Table 5:** Discriminant validity test.

	Perceived reduction in depressive symptoms (PRDS)	Emotional regulation (ER)	Perceived reduction in rumination (PRR)	Psychological resilience (PR)	AR-based fine arts education experiences (ARFAE)
Perceived Reduction in Depressive Symptoms (PRDS)	0.906				
Emotional Regulation (ER)	0.455	0.771			
Perceived Reduction in Rumination (PRR)	0.487	0.228	0.752		
Psychological Resilience (PR)	0.460	0.240	0.216	0.789	
AR-Based Fine Arts Education Experiences (ARFAE)	0.271	0.262	0.275	0.223	0.741

### 4.2 Confirmatory factor analysis

Confirmatory factor analysis was used to examine the research model, and the corresponding results are provided in **Table 6**. To determine the most appropriate factor structure, different models were compared in a systematic manner. According to the results shown in **Table 6**, the five-factor model produced the strongest overall model fit (Chi-square/df: 1.413; IFI: 0.991; SRMR: 0.030; RMSEA: 0.028; TLI: 0.988; CFI: 0.991). Therefore, the use of the five-factor model is warranted and is consistent with widely accepted research standards (92).

**Table 6:** Confirmatory factor analysis.

Fit indicators	Chi-square	df	Chi-square/df	IFI	SRMR	RMSEA	TLI	CFI
Five-factor model (ARFAE; PR; PRR; ER; PRDS)	113.050	80	1.413	0.991	0.030	0.028	0.988	0.991
Four-factor model (ARFAE+PR; PRR; ER; PRDS)	549.194	84	6.538	0.874	0.097	0.103	0.842	0.874
Three-factor model (ARFAE+PR+PRR; ER; PRDS)	1037.363	87	11.924	0.743	0.130	0.145	0.688	0.742
Two-factor model (ARFAE+PR+PRR+ER; PRDS)	1467.087	89	16.484	0.627	0.131	0.173	0.558	0.625
One-factor model (ARFAE+PR+PRR+ER+PRDS)	1731.439	90	19.238	0.556	0.148	0.188	0.479	0.554

### 4.3 Assessment of structural model

The hypotheses of the research are illustrated in **Table 7**. The findings reveal that Hypotheses 1 through 6 have been confirmed, showing significant positive correlations within these influence pathways. This conclusion aligns with academic criteria, as the z-value is greater than 1.96 and the p-value holds significance (93, 94).

**Table 7:** Research hypothesis test.

Paths			UnStd.	Std.	S.E.	Z	P	Results
AR-Based Fine Arts Education Experiences (ARFAE)	→	Perceived Reduction in Depressive Symptoms (PRDS)	0.258	0.215	0.057	4.526	***	Hypothesis 1 Effective
AR-Based Fine Arts Education Experiences (ARFAE)	→	Psychological Resilience (PR)	0.300	0.262	0.063	4.754	***	Hypothesis 2 Effective
AR-Based Fine Arts Education Experiences (ARFAE)	→	Perceived Reduction in Rumination (PRR)	0.285	0.316	0.052	5.512	***	Hypothesis 3 Effective
Psychological Resilience (PR)	→	Emotional Regulation (ER)	0.189	0.240	0.042	4.518	***	Hypothesis 4 Effective
Perceived Reduction in Rumination (PRR)	→	Emotional Regulation (ER)	0.229	0.229	0.054	4.222	***	Hypothesis 5 Effective
Emotional Regulation (ER)	→	Perceived Reduction in Depressive Symptoms (PRDS)	0.584	0.437	0.067	8.683	***	Hypothesis 6 Effective

*** represents significance with a p-value less than 0.01.

Also, the R² values for Psychological Resilience (PR), Perceived Reduction in Rumination (PRR), Emotional Regulation (ER), and Perceived Reduction in Depressive Symptoms (PRDS) were 0.534, 0.551, 0.520, and 0.573, respectively, suggesting that the model demonstrated acceptable explanatory power (95).

## 5 Summary and discussion

### 5.1 Discussion of key findings

The findings of this study should be interpreted in light of its observational, cross-sectional, and self-report design. This study does not claim that AR-based fine arts education clinically reduces, prevents, or treats adolescent depression. Rather, it shows that adolescents’ perceived AR-based fine arts education experiences were positively associated with their self-reported perceived reduction in depressive symptoms. Additionally, adolescents’ perceived AR-based fine arts education experiences were positively associated with adolescents’ psychological resilience and perceived reduction in rumination. In the hypothesized associational model, self-reported psychological resilience and perceived reduction in rumination were further positively associated with self-reported emotional regulation. Finally, self-reported emotional regulation was positively associated with adolescents’ self-reported perceived reduction in depressive symptoms. Nevertheless, the findings are broadly consistent with prior research suggesting that arts-based learning can provide adolescents with symbolic, sensory, and expressive opportunities to process emotions, construct meaning, and support psychosocial development (96).

The positive association between adolescents’ perceived AR-based fine arts education experiences and adolescents’ self-reported perceived reduction in depressive symptoms extends prior research on augmented reality in education. Existing studies have shown that AR can enhance learning engagement, interaction, visualization, and motivation by combining digital information with embodied and situated learning environments (97, 98). However, most AR education research has focused on cognitive, motivational, or usability-related outcomes rather than students’ subjective mental health-related perceptions. This study therefore contributes to this literature by suggesting that when AR is embedded in fine arts education, its immersive, interactive, and multisensory affordances may be associated with adolescents’ emotional expression, self-reflection, and perceived emotional relief.

The roles of psychological resilience, perceived reduction in rumination, and emotional regulation are also consistent with previous adolescent mental health research. Resilience has been widely regarded as a protective psychological resource that helps young people adapt to stress and adversity (27, 53, 99), while rumination is commonly understood as a maladaptive cognitive process associated with depressive symptoms (45). The present findings align with this literature by showing that adolescents’ perceived AR-based fine arts education experiences were associated with higher resilience and greater perceived reduction in rumination. One cautious interpretation is that immersive art-making activities may allow adolescents to externalize negative emotions, redirect attention from repetitive negative thinking, and experience a sense of agency through creative production. However, given the cross-sectional and self-report nature of the data, these interpretations should be understood as theoretically informed explanations rather than evidence of causal psychological mechanisms.

Beyond the observed associations, these findings may also be interpreted through the lens of experiential learning and embodied cognition perspectives (100, 101). AR-based fine arts education typically involves multimodal sensory engagement, spatial interaction, and active creation, which may be related to deeper cognitive-emotional engagement than traditional instructional formats. Within such immersive contexts, adolescents may perceive visual and artistic representation as a way to express or organize internal emotional experiences, which may be associated with lower the salience of repetitive negative thought patterns at a subjective level. At the same time, the interactive and exploratory nature of AR environments may be perceived as supporting a sense of agency and mastery, which are closely linked to psychological resilience and adaptive coping processes in adolescent development research.

Finally, the finding that emotional regulation was associated with adolescents’ self-reported perceived reduction in depressive symptoms is consistent with emotional regulation theory and related empirical evidence. Gross (102) argued that emotional regulation is central to how individuals monitor, evaluate, and modify emotional responses, and subsequent research has shown that maladaptive emotional regulation strategies are closely related to depressive and anxiety symptoms (103). This study aligns with this literature by identifying emotional regulation as a theoretically relevant correlate within the proposed relational model linking resilience and perceived reduction in rumination with adolescents’ self-reported perceived reduction in depressive symptoms. It should also be noted that this study did not test emotional regulation as a mediating factor and does not provide causal, clinical, or intervention-based evidence. Overall, this study does not provide causal, clinical, or intervention-based evidence. Instead, it offers a theoretically supported associational model showing how adolescents’ perceived AR-based fine arts education experiences are associated with adolescents’ self-reported psychological resources, perceived relief from ruminative thinking, emotional regulation, and self-reported perceived reduction in depressive symptoms.

### 5.2 Theoretical implications

This study contributes to the literature by developing and empirically examining a theoretically grounded model that links adolescents’ perceived AR-based fine arts education experiences with their perceived reduction in depressive symptoms. Rather than positioning AR-based fine arts education as a clinical intervention or claiming causal treatment effects, this study conceptualizes it as a technology-enhanced art-learning experience that may be meaningfully associated with adolescents’ self-reported psychological resources, cognitive-emotional processes, and subjective perceptions of reduced depressive symptoms. Given the observational survey design and the use of structural equation modeling, the theoretical value of the study lies not in demonstrating intervention efficacy, but in clarifying a coherent pattern of associations among perceived AR-based fine arts education experiences, self-reported psychological resilience, self-reported perceived reduction in rumination, self-reported emotional regulation, and self-reported perceived reduction in depressive symptoms.

First, this study extends theoretical discussions on the relationship between fine arts education and adolescent mental health. Prior research on arts education has often emphasized creative expression, aesthetic engagement, emotional expression, and classroom participation as important contributors to students’ psychosocial development. However, less attention has been paid to how immersive and interactive digital technologies, such as augmented reality, may reshape adolescents’ art-learning experiences and how such experiences are associated with mental health-related perceptions. By focusing on adolescents’ perceived AR-based fine arts education experiences, this study broadens the conceptualization of art education from a traditional expressive or aesthetic activity to a technologically mediated, interactive, and immersive learning context. In doing so, it positions AR not merely as an instructional tool, but as part of a broader educational experience that may be connected to adolescents’ self-reported psychological resources and emotional self-perceptions.

Second, this study contributes by integrating psychological resilience and perceived reduction in rumination into the theoretical framework linking AR-based fine arts education experiences with perceived reduction in depressive symptoms. The empirical findings suggest that adolescents’ positive experiences with AR-based fine arts education are associated with higher self-reported psychological resilience and greater perceived reduction in rumination. This extends existing theory by suggesting that the psychological relevance of technology-enhanced art education may not be limited to increased motivation, engagement, or enjoyment. Instead, such experiences may also be linked to internal adaptive resources and cognitive-emotional patterns. Psychological resilience reflects adolescents’ capacity to adapt to stress, adversity, and negative emotional experiences, whereas perceived reduction in rumination captures adolescents’ subjective sense of disengaging from repetitive negative thinking. By incorporating both constructs, the study provides a more nuanced account of how educational experiences may be associated with adolescents’ perceived emotional improvement.

Third, the study highlights emotional regulation as a theoretically relevant construct associated with psychological resources, cognitive patterns, and perceived mental health improvement. The findings indicate that self-reported psychological resilience and perceived reduction in rumination are associated with emotional regulation, which was also associated with adolescents’ perceived reduction in depressive symptoms. This offers a coherent psychological interpretation of the observed associations: AR-based fine arts education experiences, as immersive and expressive learning contexts, are associated with adolescents’ resilience and perceived reduction in rumination; these psychological and cognitive factors are then related to better emotional regulation; and emotional regulation is further associated with adolescents’ subjective perception that their depressive symptoms have decreased. Thus, this study moves beyond a simple direct association between AR-based art education and depressive symptom reduction, and instead proposes a more refined associational framework involving resilience, rumination, and emotional regulation. However, this framework should not be interpreted as evidence of mediation, temporal ordering, or causal psychological mechanisms.

Fourth, this study advances theoretical integration across educational psychology, technology-enhanced learning, arts education, and adolescent mental health research. Much of the existing AR education literature has focused on cognitive learning outcomes, motivation, spatial understanding, engagement, or usability. In contrast, this study places AR-based fine arts education within a psychological framework that emphasizes self-reported factors, such as: resilience, rumination, emotional regulation, and perceived depressive symptom reduction. By doing so, it suggests that technology-enhanced art-learning environments may have psychosocial significance beyond conventional academic outcomes. The proposed model therefore offers a foundation for future research to examine whether and how AR-supported arts education experiences may be connected to adolescents’ emotional development and subjective mental health perception.

In summary, the theoretical contribution of this study is not that it proves AR-based fine arts education can directly prevent or treat adolescent depression. Rather, it proposes and empirically examines an integrated relational model showing that adolescents’ perceived AR-based fine arts education experiences are meaningfully associated with self-reported psychological resilience, self-reported perceived reduction in rumination, self-reported emotional regulation, and self-reported perceived reduction in depressive symptoms. This framework enriches current understanding of how digital technology, artistic learning, and adolescent mental health may intersect, while also offering a rigorous theoretical basis for future causal and longitudinal investigations.

### 5.3 Practice and management implications

Given the cross-sectional, self-reported, and observational nature of the data, the following practical and managerial implications should be interpreted as exploratory suggestions based on statistical associations among adolescents’ perceived AR-based fine arts education experiences, self-reported psychological resilience, self-reported perceived reduction in rumination, self-reported emotional regulation, and self-reported perceived reduction in depressive symptoms, rather than as evidence of therapeutic or causal effects.

First, for schools and educational administrators, the findings suggest that the value of AR-based fine arts education may extend beyond artistic skill development, technological novelty, or classroom engagement. The immersive, interactive, and visual features of AR may be experienced by adolescents as offering opportunities for emotional expression, self-reflection, and meaning-making. In designing AR-supported fine arts curricula, schools may therefore consider incorporating activities that help students visualize emotions, reflect on personal experiences, and explore sources of stress and support in a safe educational environment. However, such practices should not be justified on the assumption that AR-based fine arts education has been shown to produce psychological improvement. They should not be framed as clinical treatment. Rather, they should be understood as educationally grounded approaches that may be compatible with students’ emotional development and psychosocial adjustment.

Second, curriculum designers and fine arts teachers may use the findings as a preliminary reference when developing AR-based learning activities that are sensitive to psychological resilience, perceived reduction in rumination, and emotional regulation. For example, AR art tasks could invite students to create visual representations of themes such as “coping with challenges,” “the colors of emotion,” “my future self,” or “sources of support.” Reflection prompts following these activities may help students reflect on repetitive negative thinking, identify personal strengths, and consider adaptive ways of responding to emotional experiences. Nevertheless, the present study cannot determine whether such activities actually reduce rumination, strengthen resilience, or improve emotional regulation over time. The construct of perceived reduction in rumination should be understood only as students’ self-reported perception of reduced repetitive negative thinking. Importantly, these activities should be positioned as part of social-emotional learning and well-being-oriented education, rather than as clinical interventions for depression.

Third, from a managerial perspective, the findings point to the importance of interdisciplinary collaboration if schools choose to explore AR-supported fine arts activities within broader well-being initiatives. If AR-based fine arts education is integrated into school well-being initiatives, it should not be developed solely by technology providers or art teachers. Instead, it should involve collaboration among fine arts educators, school counselors, mental health educators, educational technologists, and administrators. Fine arts educators can ensure the artistic and expressive quality of the activities; mental health professionals can advise on emotionally safe content and referral procedures; technology specialists can address usability, accessibility, and data protection; and school administrators can coordinate resources, staff training, and implementation guidelines. This collaborative model can help embed AR-based fine arts education within a broader student support system rather than treating it as a stand-alone technological innovation.Fourth, schools should pay particular attention to ethics, student safety, and individual differences when implementing AR-based fine arts activities. Because the present study concerns psychological variables related to adolescent well-being, schools should avoid over-psychologizing or labeling students based on their participation in AR art activities. Students’ artistic outputs or platform behaviors should not be used to diagnose depression, infer mental health status, or predict psychological risk. If data are collected, they should be used primarily for educational feedback, curriculum improvement, and general student support, and should be governed by principles of informed consent, parental or guardian approval, privacy protection, data minimization, and secure storage. For students who show signs of serious psychological distress, schools should have clear referral pathways to qualified counseling or clinical services.

Fifth, educational institutions may consider piloting AR-based fine arts programs on a small, low-risk, and carefully evaluated basis. These pilots should be described as educational well-being initiatives rather than depression-treatment or depression-prevention interventions. Administrators may evaluate such programs through student feedback, teacher observations, classroom engagement indicators, and self-reported measures of learning experience and well-being. However, if institutions aim to evaluate whether AR-based fine arts education produces changes in resilience, rumination, emotional regulation, or depressive symptoms, more rigorous longitudinal, quasi-experimental, or randomized controlled designs will be needed. The present findings can guide the design of such future evaluations, but they do not by themselves establish intervention efficacy. In particular, future evaluations should distinguish between perceived reduction in rumination and objectively or longitudinally measured changes in rumination.

Sixth, for mental health professionals, this study suggests opportunities for collaboration with educators rather than immediate clinical adoption of AR-based fine arts education as a therapeutic tool. Within school mental health services, AR-supported art activities may serve as complementary resources for emotional expression, psychoeducation, or social-emotional learning. They should not replace professional assessment, counseling, or treatment. Mental health professionals can play an important role in helping educators select developmentally appropriate themes, manage emotionally sensitive classroom discussions, and identify situations in which students may need additional support or referral.

Final, this study offers cautious implications for the design of future AR-supported art-learning environments. Adaptive AR platforms, personalized learning systems, and machine-learning-supported feedback may eventually contribute to more responsive art-learning environments. However, the use of algorithmic tools in adolescent mental health contexts raises significant concerns regarding privacy, validity, bias, misclassification, and ethical oversight. Therefore, any future integration of machine learning or emotion-sensitive technologies into AR-based fine arts education should be subject to rigorous ethical review, technical validation, and psychometric evaluation. This study does not provide evidence that AR systems can detect, predict, monitor, or reduce adolescents’ psychological symptoms in real time.In conclusion, the practical and managerial contribution of this study is that it identifies adolescents’ perceived AR-based fine arts education experiences as a potentially supportive educational context statistically associated with adolescents’ self-reported perceived reduction in depressive symptoms, as well as with self-reported psychological resilience and self-reported perceived reduction in rumination; self-reported psychological resilience and self-reported perceived reduction in rumination were further associated with self-reported emotional regulation, which was also associated with self-reported perceived reduction in depressive symptoms. For practitioners, this suggests the value of designing AR-supported art activities that encourage emotional expression, reflection, and adaptive coping within school-based well-being frameworks. For managers and administrators, it highlights the need for interdisciplinary collaboration, ethical implementation, teacher training, data protection, and referral mechanisms. However, until longitudinal and intervention-based evidence is available, AR-based fine arts education should not be described as a clinical effective treatment or prevention strategy for adolescent depression. Rather, it should be viewed as a potentially supportive educational resource whose mental health relevance requires further empirical validation.

## 6 Research limitations

Although this study provides meaningful initial evidence regarding the relationships between AR-based fine arts education experiences and adolescent psychological variables, several limitations should be acknowledged. First, this study employed a cross-sectional survey design and structural equation modeling. Therefore, the findings should be interpreted as associational evidence rather than causal evidence. Although the hypothesized relationships were empirically tested using structural equation modeling, cross-sectional SEM is best suited to examining theoretically specified patterns of covariance among constructs, rather than establishing temporal precedence or causal change. Accordingly, the observed paths among perceived AR-based fine arts education experiences, self-reported psychological resilience, self-reported perceived reduction in rumination, self-reported emotional regulation, and self-reported perceived reduction in depressive symptoms should be understood as statistical associations within a theoretically informed model. Future studies using longitudinal, quasi-experimental, or randomized controlled designs would be valuable for examining whether these associations reflect temporal or causal processes.

Second, the sample was drawn from Chinese adolescents. Although the sample size of 518 participants provides a reasonable empirical basis for testing the proposed model, the cultural and educational context of the sample should be considered when interpreting the findings. Adolescents in China may differ from those in other countries in terms of school context, family expectations, educational experiences, curriculum structure, and access to digital technologies. Therefore, the generalizability of the findings to other cultural, educational, or socioeconomic settings should be examined in future research. Cross-cultural and multi-site studies would help determine whether the proposed model is stable across different adolescent populations and learning environments.

Third, this study relied on self-reported survey data. Self-report measures are appropriate for capturing adolescents’ subjective educational experiences and perceived psychological states, which were central to the aims of this study. However, they may also be influenced by common method variance, social desirability bias, recall bias, or individual response tendencies. This issue is particularly relevant because the core constructs in the model, including perceived AR-based fine arts education experiences, self-reported psychological resilience, self-reported perceived reduction in rumination, self-reported emotional regulation, and self-reported perceived reduction in depressive symptoms, were assessed through adolescents’ subjective reports. In particular, perceived reduction in rumination refers to adolescents’ self-reported perception that their repetitive negative thinking was reduced, rather than an objectively observed or longitudinally verified reduction in rumination. Future research could strengthen measurement robustness by combining self-reports with multi-informant or multi-method data, such as teacher assessments, parent reports, classroom observations, behavioral tasks, platform-based learning records, or physiological indicators.

Fourth, this study did not include demographic or contextual control variables in the structural equation model. Future studies could incorporate relevant covariates or conduct subgroup and multi-group analyses to examine whether the proposed relationships vary across gender, age, grade level, socioeconomic background, urban-rural context, prior exposure to AR technology, baseline mental health status, or previous arts education experience. Such analyses would help clarify whether the observed associations are broadly consistent across adolescent groups or are more pronounced in particular subpopulations.

Fifth, although the three-item scales used for each variable demonstrated acceptable reliability and validity, they provide limited psychometric resolution and may not fully capture the multidimensional nature of perceived AR-based fine arts education experiences and related psychological constructs. Future studies should develop more comprehensive instruments. Finally, it is important to note that the dependent variable in this study was adolescents’ self-reported perceived reduction in depressive symptoms, rather than clinically diagnosed depression or objectively verified symptom improvement. Therefore, the findings should be interpreted as evidence of associations between perceived AR-based fine arts education experiences and adolescents’ subjective perceptions of psychological improvement, rather than as evidence of a clinical treatment effect. Similarly, because the study did not include baseline and follow-up assessments, it cannot determine whether depressive symptoms or rumination changed over time. Future studies that incorporate clinical screening, repeated symptom tracking, validated rumination measures, diagnostic procedures, or professional assessments would be useful for examining the mental health implications of AR-based fine arts education more rigorously.

Overall, these limitations suggest that the findings should be interpreted with appropriate caution, but they do not diminish the study’s contribution as an initial empirical examination of an emerging educational and psychological phenomenon. The present research offers a theory-informed associational model showing that adolescents’ perceived AR-based fine arts education experiences covary with self-reported psychological resilience, self-reported perceived reduction in rumination, self-reported emotional regulation, and self-reported perceived reduction in depressive symptoms. By identifying these associations, the study provides a useful empirical foundation for future longitudinal, experimental, multi-informant, and clinically informed research on AR-supported fine arts education and adolescent well-being.

## Data Availability

The datasets presented in this article are not readily available due to the involvement of minors and the inclusion of mental-health-related responses, the raw data supporting the findings of this study are not publicly available in order to protect participants’ privacy and confidentiality. De-identified data may be made available from the corresponding author upon reasonable request, subject to ethical approval, institutional regulations, and applicable privacy protection requirements. Requests to access the datasets should be directed to Chengrui Gao, cr@stu.scu.edu.cn.
